# Two novel ligand-independent variants of the VEGFR-1 receptor are expressed in human testis and spermatozoa, one of them with the ability to activate SRC proto-oncogene tyrosine kinases

**DOI:** 10.18632/oncotarget.27232

**Published:** 2019-10-08

**Authors:** Belen Alvarez-Palomo, Carme Barrot-Feixat, Helena Sarret, Jordi Requena, Montserrat Pau, Jose-Manuel Vidal-Taboada, Rafael Oliva, Josep-Lluis Ballesca, Michael J. Edel, Jovita Mezquita-Pla

**Affiliations:** ^1^Molecular Genetics and Control of Pluripotency Laboratory, Department of Biomedicine, Biomedical Research Institute August Pi i Sunyer (IDIBAPS), Institute of Neurosciences, Faculty of Medicine and Health Sciences, University of Barcelona, Barcelona, Catalonia, Spain; ^2^Forensic Genetics Laboratory, Medicine Department, Faculty of Medicine and Health Sciences, University of Barcelona, Barcelona, Catalonia, Spain; ^3^Molecular Genetics Laboratory, Department of Biomedicine, Faculty of Medicine and Health Sciences, University of Barcelona, Barcelona, Catalonia, Spain; ^4^Peripheral Nervous System, Neuroscience Department, Vall d’Hebron Research Institute (VHIR), Barcelona, Catalonia, Spain; ^5^Molecular Biology of Reproduction and Development Laboratory, Biomedical Research Institute August Pi i Sunyer (IDIBAPS), Department of Biomedicine, Faculty of Medicine and Health Sciences, University of Barcelona, Barcelona, Catalonia, Spain; ^6^Biochemistry and Molecular Genetics Service, Biomedical Diagnostic Centre, Hospital Clinic, Barcelona, Catalonia, Spain; ^7^Clinic Institute of Gynaecology, Obstetrics and Neonatology, Hospital Clinic, Barcelona, Catalonia, Spain; ^8^International Research Fellow, Victor Chang Cardiac Research Institute, Sydney, New South Wales, Australia; ^9^Senior Research Fellow, University of Western Australia, School of Medicine and Pharmacology, Harry Perkins Research Institute Centre for Cell Therapy and Regenerative Medicine (CCTRM), Perth, Western Australia, Australia; ^*^First authors

**Keywords:** ligand-independent intracellular iVEGFR-1 isoforms, mature testis, spermiogenesis, SRC activation, fertility

## Abstract

The vascular endothelial growth factor receptor 1 (VEGFR-1) family of receptors is preferentially expressed in endothelial cells, with the full-length and mostly the soluble (sVEGFR-1) isoforms being the most expressed ones. Surprisingly, cancer cells (MDA-MB-231) express, instead, alternative intracellular VEGFR-1 variants. We wondered if these variants, that are no longer dependent on ligands for activation, were expressed in a physiological context, specifically in spermatogenic cells, and whether their expression was maintained in spermatozoa and required for human fertility. By interrogating a human library of mature testis cDNA, we characterized two new truncated intracellular variants different from the ones previously described in cancer cells. The new isoforms were transcribed from alternative transcription start sites (aTSS) located respectively in intron-19 (i_19_VEGFR-1) and intron-28 (i_28_VEGFR-1) of the VEGFR-1 gene (GenBank accession numbers JF509744 and JF509745) and expressed in mature testis and spermatozoa. In this paper, we describe the characterization of these isoforms by RT-PCR, northern blot, and western blot, their preferential expression in human mature testis and spermatozoa, and the elements that punctuate their proximal promoters and suggest cues for their expression in spermatogenic cells. Mechanistically, we show that i_19_VEGFR-1 has a strong ability to phosphorylate and activate SRC proto-oncogene non-receptor tyrosine kinases and a significant bias toward a decrease in expression in patients considered infertile by WHO criteria.

## INTRODUCTION

During meiotic and post-meiotic stages of spermatogenesis, the genome is reprogrammed prior to packaging. Simultaneously, new mRNAs responsible for active and precise synthesis of proteins are transcribed with stage specific precision. Selected transcripts and proteins, essential for spermatozoa activity, fertilization or embryo development are retained in the spermatozoa [1, 2].

Many spermatogenic transcripts are produced from alternative transcription start sites and alternative splicing of the 5′UTR. Although these mRNAs may code for proteins identical to their somatic counterparts, they often differ, from the corresponding somatic transcripts, in regulatory potential [3, 4].

Protein tyrosine kinases (PTKs) are key factors regulating spermatogenesis, essential for differentiation, migration, adhesion, spermatid shaping, motility and capacitation. In addition to the general requirement for PTKs, normal spermatogenesis and spermiogenesis also seem to be dependent on the expression of truncated isoforms [5]. Truncated proteins, many of them protein kinases, either coexist with full-length partners or replace them. For example, the KIT proto-oncogene receptor tyrosine kinase is highly expressed in spermatogonia and is replaced by the truncated-intracellular KIT version (Tr-KIT) during spermiogenesis [6–9]. Interestingly, there is a high degree of similarity between VEGFR-1 and KIT, and between i_19_VEGFR-1 and Tr-KIT.

Vascular endothelial growth factor receptor 1 (VEGFR-1), also known as FLT-1 (fms related tyrosine kinase 1) is a high-affinity and low-activity (relatively to VEGFR-2) tyrosine kinase receptor of the vascular endothelial growth factor VEGF-A, and binds selectively to ligands VEGF-B and placental growth factor (PlGF) [10–13]. Expression of the VEGFR-1 receptor occurs mainly in endothelial cells, but also takes place in different types of non-endothelial cells [14, 15], including mouse spermatids and spermatozoa [16].

VEGFR-1 in humans consists of 30 exons spanning more than 193 kb [17]. Two extracellular soluble isoforms, sVEGFR-1 and s14VEGFR-1, end in exons generated by extending into intron 13 (sVEGFR-1) or jumping into intron 14 (s14VEGFR-1) [18, 19]. In addition, we have characterized several intracellular isoforms of VEGFR-1 expressed in cancer cells [20].

In mouse [16], the vascular endothelial growth factor receptor 2 (VEGFR-2), the high-activity and low-affinity receptor for VEGF-A, comparatively to VEGFR-1 (also known as KDR, kinase insert domain receptor) is expressed in type A spermatogonia, while VEGFR-1 is not expressed until the pachytene spermatocyte and round spermatid stages. There is no knowledge of the specific VEGFR-1 isoforms expressed in mouse spermatogenic cells, and contrary to human [20], no truncated isoforms of the VEGFR-1 receptor are known to be expressed.

In this paper, we have identified two intracellular isoforms of the VEGFR-1 receptor in mature testis and spermatozoa. We have named them i_19_VEGFR-1 and i_28_VEGFR-1, as they start transcription in intron 19 and 28, respectively, and we have characterized them by sequencing, RT-PCR, northern and western blot. Mechanistically, we have demonstrated, using binding experiments and transfection of recombinant plasmids in CHO and HEK293 cells, that i_19_VEGFR-1 is able to bind and strongly phosphorylate and activate SRC protein kinases.

As a preliminary study, due to sample scarcity and the lack of confident parameters for fertility, we have analyzed by Real time-PCR, the expression of both isoforms in samples of astenozoospermic, oligozoospermic or oligoastenozoospermic patients relative to normozoospermic samples (WHO parameters) [21]. We wanted to assess the predictive potential of their expression as a marker for male infertility, a major problem worldwide because of inaccurate diagnosis, much dependent on empirical evidence and unreliable parameters.

We have also explored by an *in silico* approach, taking advantage of the huge quantity of available data in well-documented networks and testis cell lines, the chromatin landscape and the response element present in the corresponding proximal promoters around the TSS, of both isoforms. The reported results showed a relatively open chromatin configuration, punctuated by activating marks signatures and regulatory DNA sequence elements, which may allow for transcription in post-meiotic cells.

Taken together, the two i_19_VEGFR-1 and i_28_VEGFR-1 isoforms described in this paper may represent haploid cell specifically expressed transcripts that function as convenient ligand-independent intracellular factors with interest in fertility and fertilization.

## RESULTS

### Identification of two novel intracellular truncated C-terminal isoforms of VEGFR-1

**Figure 1:**
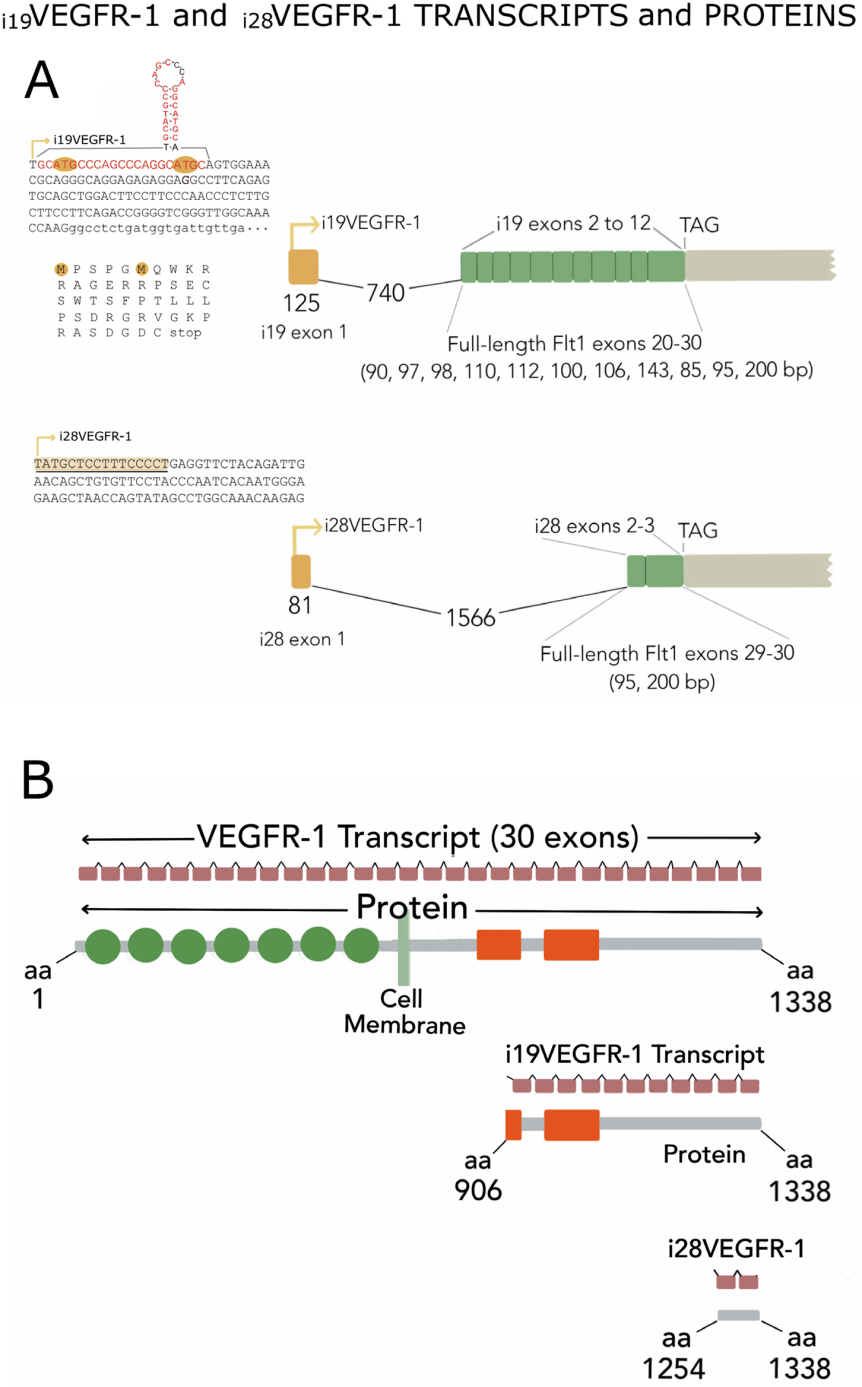
(**A**) Exon assembly of i_19_VEGFR-1 and i_28_VEGFR-1 isoforms indicating start of transcription (arrows), specific first exon (yellow) and intron 1 (broken line with nucleotide’s number), followed by the exons shared with the full-length receptor, and the stop codon (TAG). Top left, whole exon 1 (capital letters) and first 22 nucleotides of exon 2 (lower case) of i_19_VEGFR-1. Exon 1 starting with the p53 sequence element (in red), is depicted with the putative hairpin structure. The open reading frames (uORF) are indicated, highlighting the ATG codon, as well as the non-in-frame putative peptides in the leader 5′UTR sequence. Below, the complete first exon of i_28_VEGFR-1 with the polypyrimidine stretch (underlined) at the start of transcription (arrow). (**B**) Gene and protein assembly. The 30 exons (small brown rectangles) spanning the full- length VEGFR-1 receptor, or the eleven (i_19_VEGFR-1) and two (i_28_VEGFR-1) coding exons, are shown on top of each schematic protein. From top to bottom, full-length protein of 1,338 amino acids (aa), i_19_VEGFR-1 intracellular isoform of 432 aa, and i_28_VEGFR-1 intracellular isoform of 85 aa. Circles denote extracellular immunoglobulin domains not present in the intracellular variants; orange rectangles show the split kinase domain, or slightly trimmed kinase in i_19_VEGFR-1.

A search by Rapid amplification of cDNA ends (RACE5′ and RACE3′) for truncated intracellular isoforms of the VEGFR-1 receptor, failed to detect previously characterized intracellular isoforms [20] of the VEGFR-1 receptor in mature human testis and spermatozoa. We also interrogated a mouse mature testis cDNA library and could not find any truncated isoform of the VEGFR-1 receptor. In contrast, the same analysis, by rapid amplification of c-DNA ends (RACE), RACE5′ and RACE3′, of a mature testis human cDNA library, allowed us to obtain two novel truncated intracellular isoforms variants of the VEGFR-1 receptor. We named these intracellular isoforms i_19_VEGFR-1 and i_28_VEGFR-1, the number indicating the intron where the aTSS is located (Figure 1).

Both isoforms lack the sequences for extracellular domains, transmembrane domain and either, part of the kinase domain (i_19_VEGFR-1) or the whole kinase domain, leaving only a sequence coding for a C-terminal tail of 85 amino acids in the case of i_28_VEGFR-1. Both isoforms incorporate new leader 5′UTR sequences (Figure 1A). For the 3′UTR, we obtained a sequence of 675 nucleotides, finishing in a rich poly (A) sequence. This 3′UTR is much shorter than the canonical VEGFR-1 3′UTR observed in endothelial cells. Northern blot reinforced the predominance of this 3′UTR (as mentioned later), also showing the existence, in much lower amounts of the longer canonical 3′UTRs.

Isoform i_19_VEGFR-1 starts at nucleotide 1,200 of intron 19 of the full-length VEGFR-1 receptor, in a T nucleotide that is followed by a sequence element, where 19 nucleotides out of 21 matches the p53 sequence element [22] (red in Figure 1A). This motif can form a hairpin structure (Figure 1A). The first exon of this isoform has a conserved splice site (gt) that jumps to the second exon acceptor site (ag), generating a VEGFR-1 isoform of 12 exons, the last 11 corresponding to exons 20 to 30 of the full length VEGFR-1. The 5′UTR of the new isoform, is 129 nucleotides long, and shows two out of frame uORFs (Figure 1A).

**Figure 2:**
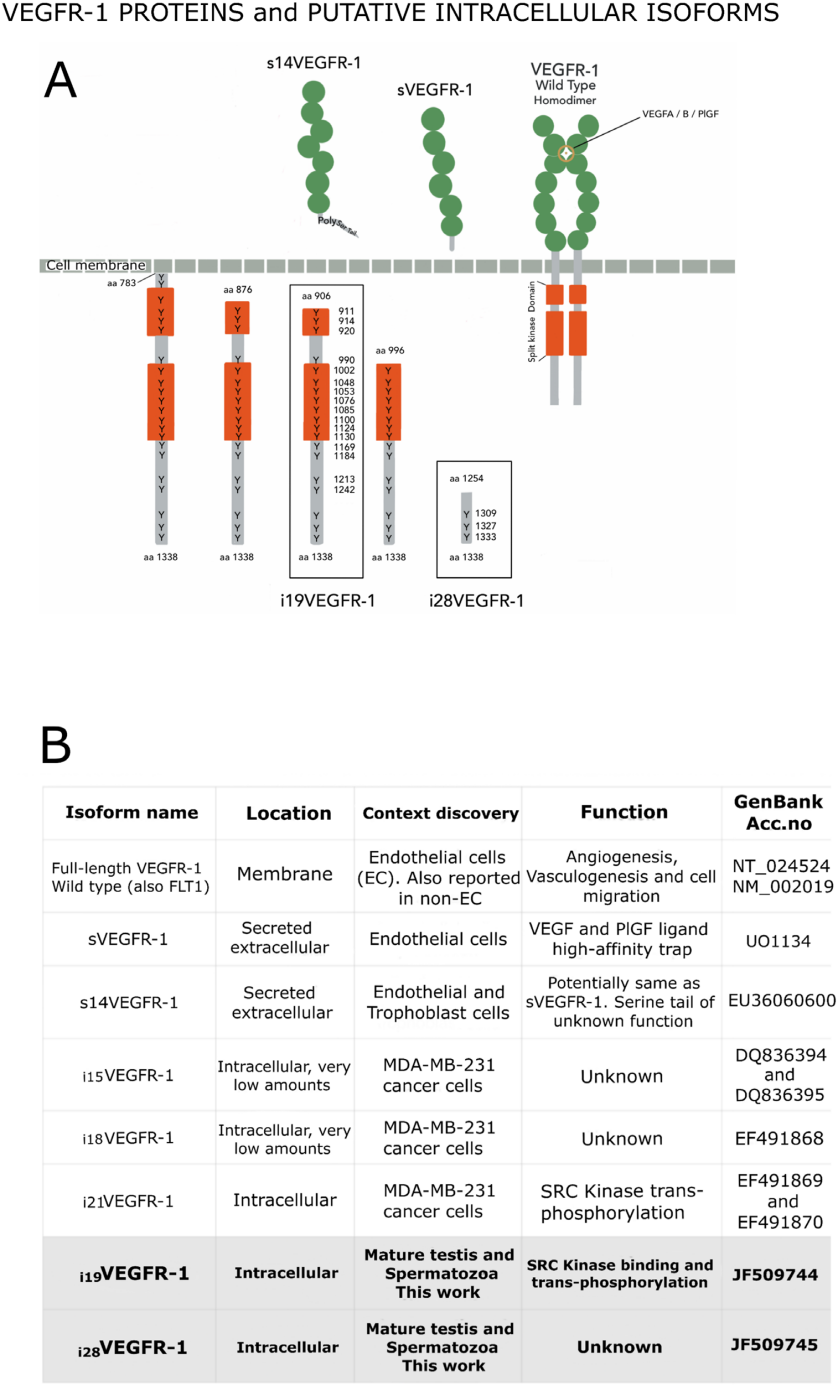
(**A**) VEGFR-1 receptor proteins shown in their respective compartments, with the numbering of their tyrosine’s residues, which are found at high density within the intracellular domain. Extracellular (named soluble) VEGFR-1 proteins contain the immunoglobulin domains (green circles) and end in specific tails. The full-length transmembrane VEGFR-1 receptor contains the immunoglobulin domains, the transmembrane and the juxta-membrane domains, as well as the intracellular split kinase domain ending in a C-terminal tail. Intracellular isoforms contain the whole kinase domain, part of it, or non-kinase domain (i_28_VEGFR-1). (**B**) Box detailing distinctive characteristics for the different VEGFR-1 isoforms.

The putative i_19_VEGFR-1 transcript encodes a protein of 433 amino acids, from amino acid 906 to amino acid 1,338 of the full-length VEGFR-1 receptor, with a predicted molecular weight of 49.4kDa; isoform i_19_VEGFR-1 conserves 253 of the 332 amino acids of the kinase domain. It has lost most of the ATP-binding domain, but it conserves the phosphotransferase site as well as eight tyrosine-phosphorylation sites (Figure 1B and Figure 2). ****

Isoform i_28_VEGFR-1 starts at nucleotide 308 of intron 28 of the full-length VEGFR-1, in a very rich polypyrimidine stretch. The first exon of this isoform has a conserved splice site (gt) that jumps to the second exon acceptor site (ag), generating a small VEGFR-1 isoform of 3 exons, the last 2 corresponding to exons 29-30 of the full-length VEGFR-1. The 5′UTR of the new isoform is 135 nucleotides long. The putative i_28_VEGFR-1 encodes a protein of 85 amino acids, from amino acid 1,254 to amino acid 1,338 of the full-length VEGFR-1 receptor, with a molecular weight of 9.5kD. The i_28_VEGFR-1 isoform lacks the entire kinase domain (Figure 1B), and conserves the last 3 phosphotyrosine sites (Figure 2).

### Expression of i_19_VEGFR-1 and i_28_VEGFR-1 isoforms in human mature testis, cryptorchidic testis, spermatozoa, and HUVEC cells, relative to the expression of canonical VEGFR-1, full-length and soluble variants, and in samples with accepted or altered WHO 2010 parameters

We analyzed the expression of i_19_VEGFR-1 and i_28_VEGFR-1 and of full-length VEGFR-1, sVEGFR-1 and s14VEGFR-1 isoforms, by northern-blot and/or semi-quantitative manual PCR, and Real-Time PCR, in human mature testis, cryptorchidic testis, HUVEC cells (a positive control for VEGFR-1), and human spermatozoa.

**Figure 3:**
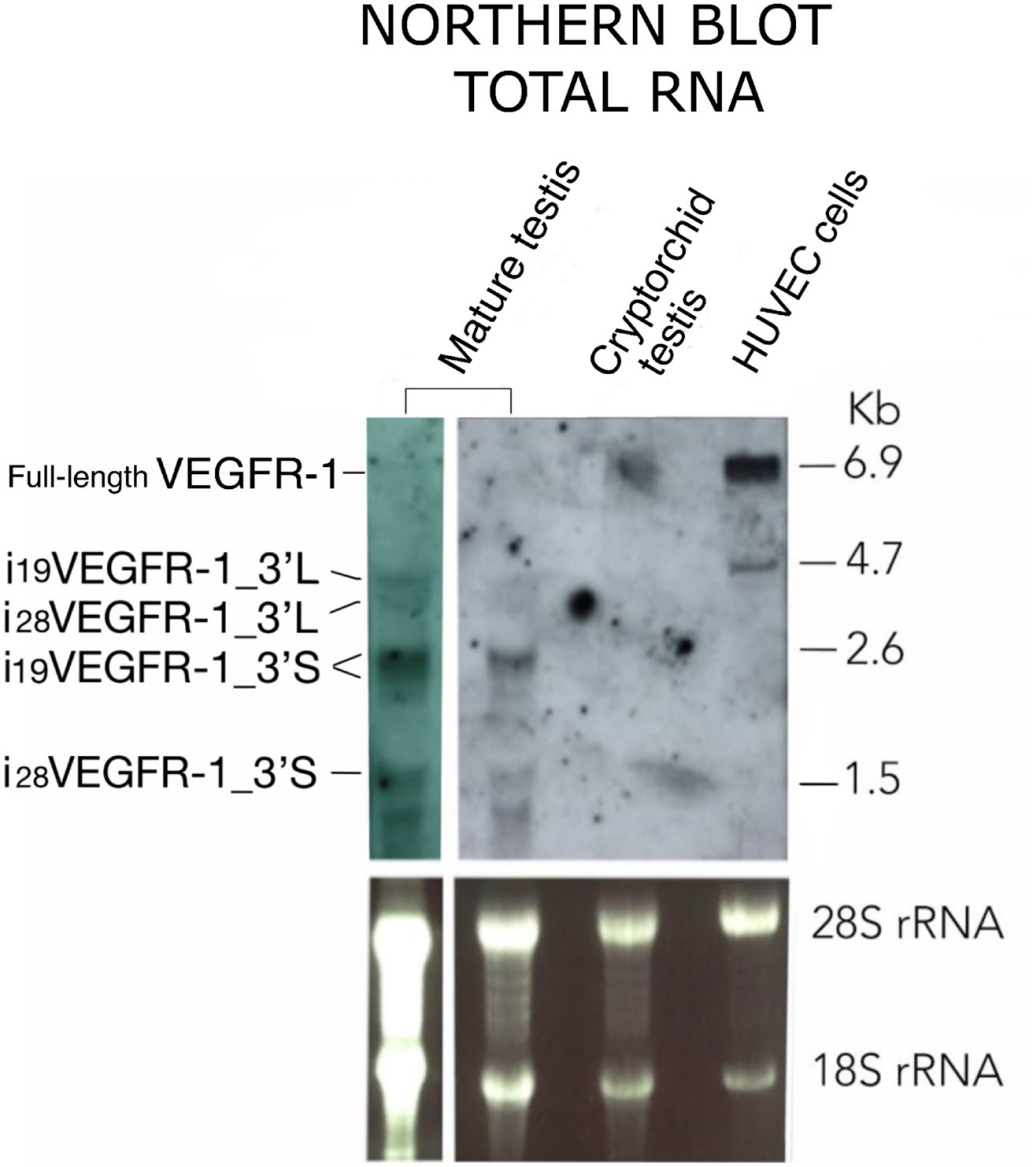
Northern blot pattern of VEGFR-1 from total RNA expressed in mature testis, cryptorchid testis and HUVEC cells as indicated. Agarose-formaldehyde gel blotted and hybridized using a probe complementary to the kinase domain. RNA loads correspond to 40 μg (left lane) or 20 μg (all other lanes) of total RNA. 3′L and 3′S denote i_19_VEGFR-1 and i_28_VEGFR-1 transcripts, displaying either, whole length or shorter (675 nt) 3′UTR, respectively. Bottom panel: Ribosomal staining as a visual indicator of sample loading.

Figure 3 shows the northern-blot results obtained from mature testis, cryptorchidic testis and endothelial HUVEC cells using total RNA and a probe from the kinase region (Table 1). The pattern of expression in testis differs entirely from endothelial cells. In HUVEC cells, full-length VEGFR-1 mRNA is detected as a band of ≈6.9kb, and a second band at ≈4.7kb, corresponds to a truncated isoform. Cryptorchidic testis, devoid of spermatogenic differentiating cells, shows only, a very faint band of ≈6.9kb. The pattern of expression in mature testis correspond to bands of ≈4.7kb, ≈2.6kb and ≈1.5kb, all of them compatible with the expected sizes of i_19_VEGFR-1 and i_28_VEGFR-1 transcript isoforms, having different 3′UTR lengths, but preferentially using the short 3′UTR of 675 nucleotides obtained by RACE3′ (3′S, Figure 3). The northern pattern results are also in accordance with the relative expression of transcripts obtained by quantitative RT-PCR.

**Table 1:** Primers, with indication of gene sequence, size, polarity and specificity

**Primer**	**Sequence**	**Size**	**Polarity/position in full-length VEGFR-1**
HFE11U	5′CATCACTCAGCGCATGGCAAT3′	21 mer	Forward/exon 11
HFE12U	5′AGCACCTTGGTTGTGGCTGAC3′	21 mer	Forward/exon12
HFs13D	5′TTGTTGCAGTGCTCACCTCTGA3′	22 mer	Reverse/intron 13 sVEGFR-1 specific/splints exon and intron 13.
HFE11U-HFs13D		474 bp	Detects sVEGFR-1. Skips intron11 and exon and intron 12.
HFE12U-HFs13D		428 bp	Detects selectively sVEGFR-1 isoform. Skips intron 12.
HFE14U	5′GATCAGGAAGCACCATACCTCCTG3′	24 mer	Forward/exon 14
HFs14D	5′GGTGATGATGACGATGACGATGG3′	23 mer	Reverse/intron 14, s_14_VEGFR-1 specific.
HFE14U- HFs14D		191 bp	Detects selectively s_14_VEGFR-1 isoform. Skips intron 14
HFe14U	5′TCCCCGAGCCTCAGATCACTTG3′	22 mer	Forward/exon 14
Flt1Fw	5′GATGTTGAGGAAGAGGAGGATT3′	22 mer	Forward/splints exons 21 and 22.
Flt1Rv	5′AAGCTAGTTTCCTGGGGGTATA3′	22 mer	Reverse/exon 30, 3′UTR
Flt1Fw-Flt1Rv		1,146 bp	Probe synthesis
HFE15D	5′CTGGTTGGTGGCTTTGCAGTG3′	21 mer	Reverse/exon 15
HFE14U-HFE15D		241 bp	Detects full-length VEGFR-1 as well as isoforms with membrane and juxta-membrane domains.
HFI19U	5′CCCAGGCATGCAGTGGAAAC3′	20 mer	Forward/intron 19, i_19_VEGFR-1 specific
HF20D	5′CGTTTGCTCTTGAGGTAGTTGGAG3′	24 mer	Reverse/exon 20
HFI19U-HF20D		190 bp	Detects selectively i_19_VEGFR-1. Skips a large fragment of intron 19.
HFE22D	5′GACAGGAACTCCATGCCTCTGG3′	22 mer	Reverse/exon 22
HFI19U-HF22D		457 bp	Detects selectively i_19_VEGFR-1. Skips a large fragment of intron 19 plus exons and introns 20 and 21.
HFI28U	5′CAGCTGTGTTCCTACCCAATCAC3′	23 mer	Forward/intron 28, i_28_VEGFR-1 specific
HFE30D	5′TCAGACAGCCCCGACTCCTTAC3′	22 mer	Reverse/exon 30
HFI28U-HFE30D		197 bp	Detects selectively i_28_VEGFR-1. Skips exon intron 29.
HFE29U	5′GACTACCAGGGCGACAGCAGC3′	21 mer	Forward/exon 29
HFE30D	5′TCAGACAGCCCCGACTCCTTAC3′	22 mer	Reverse/exon 30
HFE29U- HFE30D		137 bp	Amplifies all variants isoforms containing the C-terminal tail.
HCID	5′CAGCTGGAATGGCAGAAACTGG3′	22 mer	Reverse/exon 30
HFE29U-HCID		170 bp	Amplifies all variants isoforms containing the C-terminal tail.
HFrev2	5′GGCAAAAGCTAGTTTCCTGGGGGTATA3′	27 mer	Reverse/exon 30, 3′UTR.
19EAT	5′ATACAGAATTCGATGGTGATTGTTGAAT3′	28 mer	i_19_VEGFR-1 cloning
HFFP	5′GTCGTCATCCTTGTAATCGATGGGTGGG GTGGAG3′	34 mer	i_19_VEGFR-1 cloning plus partial flag tagging.
HFFFA	5′CAAGAGGGCCCTCACTTATCGTC GTCATCCTTGTA3′	35 mer	i_19_VEGFR-1 cloning and complete flag tagging.
HAF	5′CCTCGCCTTTGCCGATCC3′	18 mer	Forward/endogenous β-Actin
HAR180	5′ATCACGCCCTGGTGCCTG3′	18 mer	Reverse/endogenous β-Actin
HAF-HAR180		180 bp	β-Actin. Skips intron.

**Figure 4:**
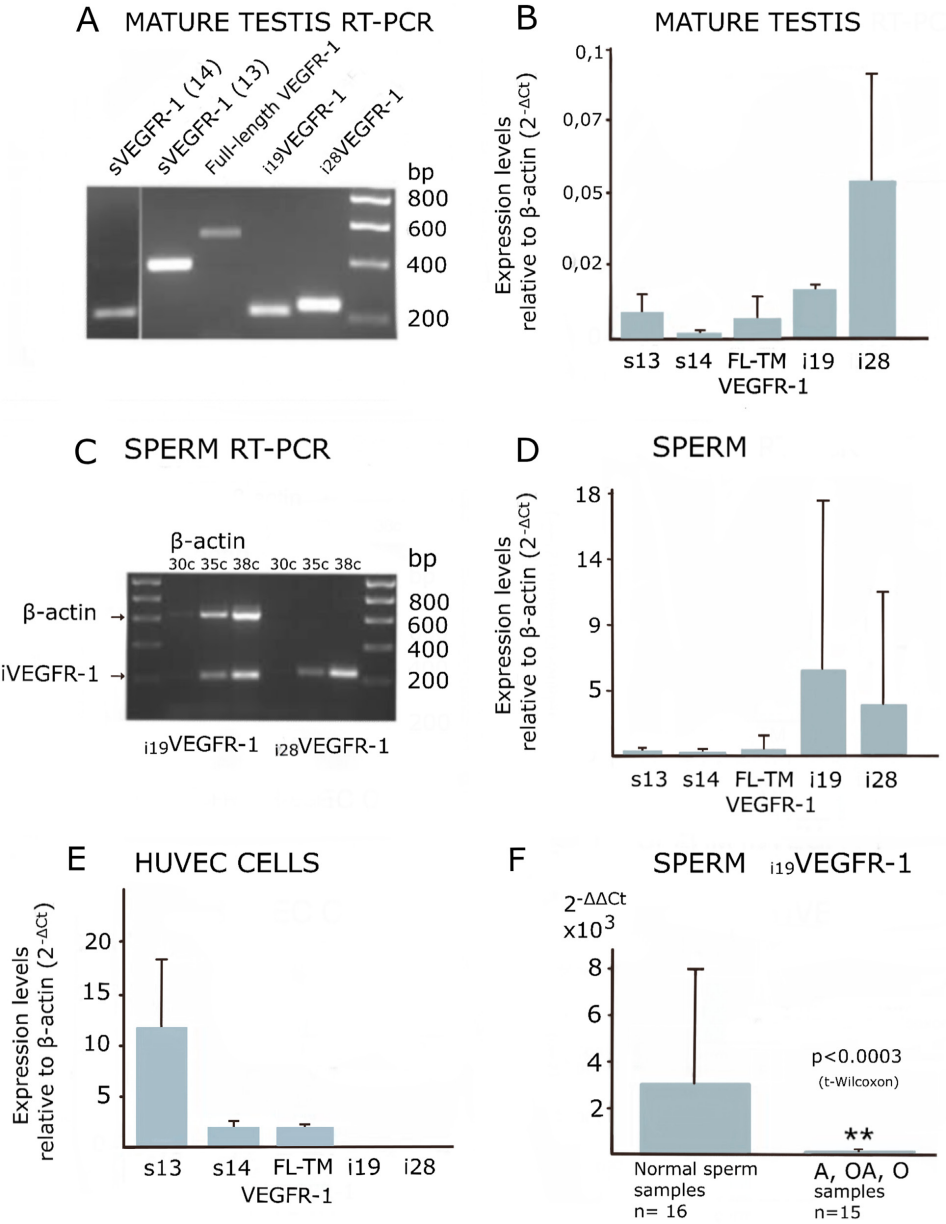
Expression of VEGFR-1 variants analyzed by manual, semi-quantitative and real-time PCR. (**A** and **C**) Ethidium bromide image of a 1.5% agarose gel. (**B**, **D** and **E**) Real time-PCR quantification relative to β-actin, showing standard deviation. (**F**) Statistical Bar Graph showing relative expression values of i_19_VEGFR-1 in spermatozoa, relative to β-actin and referred to i_19_VEGFR-1 levels in mature testis (2^-ΔΔ Ct^). The pool of sperm defective samples (see methods), *n* = 15 shows a significantly decreased (^**^ *p* < 0.0003) in i_19_VEGFR-1 expression when compared with the pool of non-defective spermatozoa (*n* = 16, normal) by the same parameters. VEGFR-1, full-length (FL) transmembrane (TM) receptor, sVEGFR-1 (s13), s14VEGFR-1 (s14).

By semi-quantitative, as well as by Real-Time PCR, we detected significant levels of i_19_VEGFR-1 and i_28_VEGFR-1 transcripts only in mature testis and spermatozoa (Figure 4A–4D). Full-length and both soluble forms, sVEGFR-1 and s14VEGFR-1, were also detected by PCR amplification in testis and spermatozoa, although at levels much lower than in HUVEC cells (Compare Figure 4B and 4D to 4E).

Isoforms i_19_VEGFR-1 and i_28_VEGFR-1, as detected by Real-Time PCR, showed higher expression in spermatozoa than in testis, suggesting preferential expression in haploid spermatogenic cells. (Compare Figure 4B with Figure 4D).

Quantitative real-time PCR of i_19_VEGFR-1 and i_28_VEGFR-1 mRNA from spermatozoa obtained from astenozoospermic, oligozoospermic or oligoastenozoospermic semen samples revealed statistically significant lower levels of i_19_VEGFR-1 expression compared to normozoospermic semen samples (Figure 4F). Expression of i_28_VEGFR-1 was also investigated but, although there was a tendency towards decreased levels in infertile samples, they were no statistically significant (data not shown).

Western blotting was employed to determine whether i_19_VEGFR-1 and i_28_VEGFR-1 transcripts were translated into proteins.

**Figure 5:**
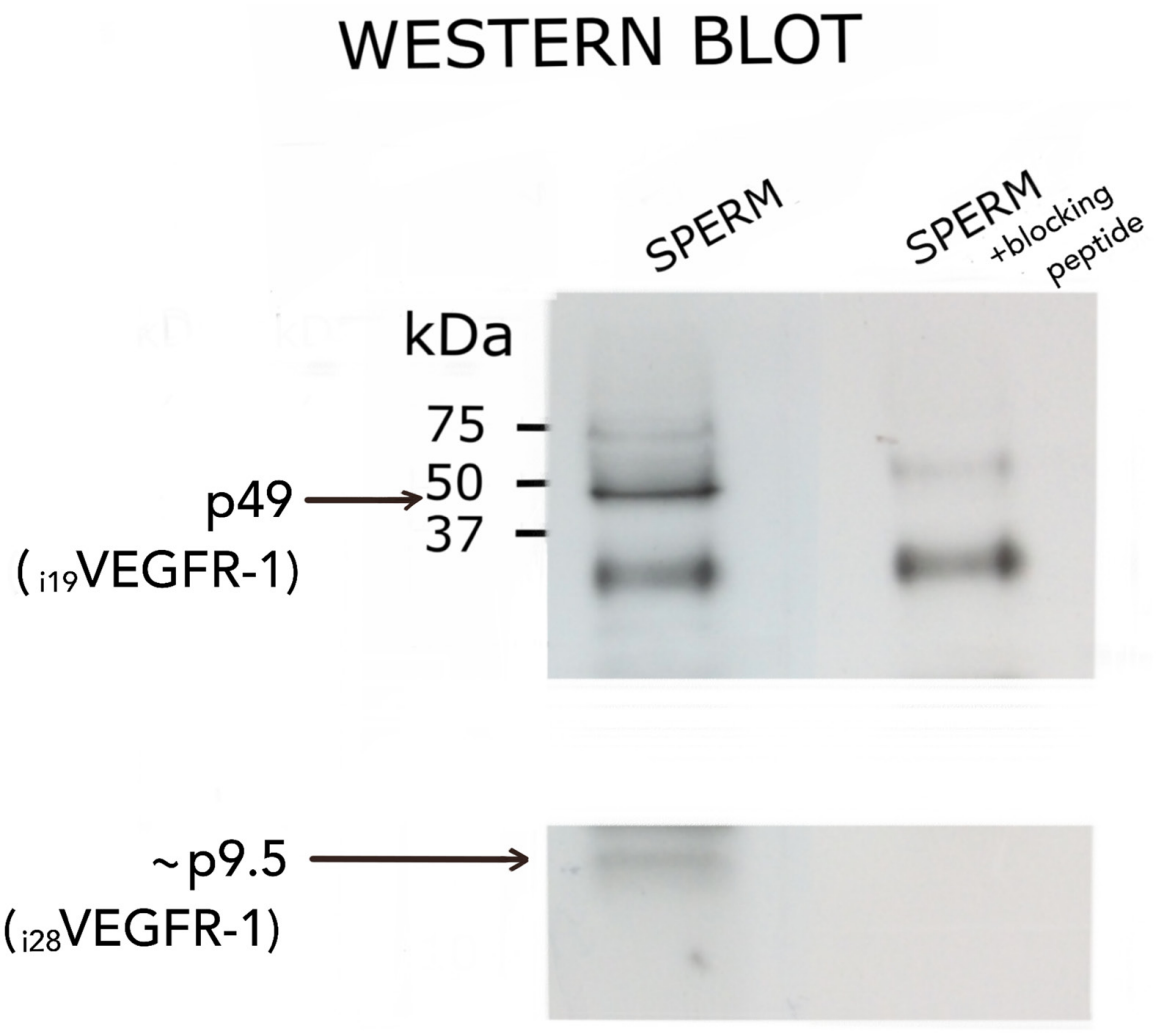
Western blot analysis of i_**19**_VEGFR-1 (p49) (49kDa) and putative i_**28**_VEGFR-1 (p9.5) (9.5kDa) isoforms expressed in normal sperm samples. Detection was carried out with the anti-VEGFR-1 antibody sc-316 from Santa Cruz, and validated by competition with a specific peptide (see methods). At left, molecular weight markers BioRad. The cropped part of the blot correspond to a disturbing unspecific artifact present in both, competed and non-competed lines.

Figure 5 shows the protein corresponding to the i_19_VEGFR-1 isoform. It was detected in spermatozoa using an anti-VEGFR-1 antibody from the C-terminal of the protein (Santa Cruz, see materials and methods and Table 2). Binding of the antibody at the corresponding size of 49kD competed with an epitope specific blocking peptide (Santa Cruz) from the same region indicating the specificity of the western blot bands shown in Figure 5. The peptide corresponding to _i28_VEGFR-1, expected as a 9.5kD band, was not as consistently detected. It appears as a faint band suggestive of a quite unstable peptide. The cropped part of the blot correspond to a disturbing unspecific artifact present in both, competed and non-competed lines.

**Table 2:** Antibodies used

**Primary Antibodies**	**Host species**	**Reactivity/cross-reactivity**	**Dilution**	**Company**	**Clonality**
Anti-VEGFR-1 #2893	Rabbit	Human/do not cross-react with VEGFR-2 or VEGFR-3, neither with sVEGFR-1	1:1000	Cell Signaling	Polyclonal against residues surrounding Thr 1307
Src #2108	Rabbit	Human and other species	1:1000	Cell Signaling	Polyclonal against residues close to the carboxylate terminus of human Src
Phospho-Src (Tyr 418) # 44-660G, RRID AB_2533714	Rabbit	Human and other species	1:1000	Invitrogen/ThermoFisher Scientific	Polyclonal against P^Y418^
Flt-1 C-17 #Sc-316	Rabbit	Human and other species	1:200	Santa Cruz Biotechnology	Polyclonal against C-terminus
Fyn3 #sc16	Rabbit	Human and other species	1:300	Santa Cruz Biotechnology	Polyclonal
Anti-β Actin #A5316	Mouse	Human and other species	1:5000	Sigma	Monoclonal
Anti α-Tubulin #T6074	Mouse	Human and other species	2 ng/mL	Sigma	Monoclonal epitope at C-terminal

### Protein kinase receptor i_19_VEGFR-1 binds and activates Src-protein kinases

**Figure 6:**
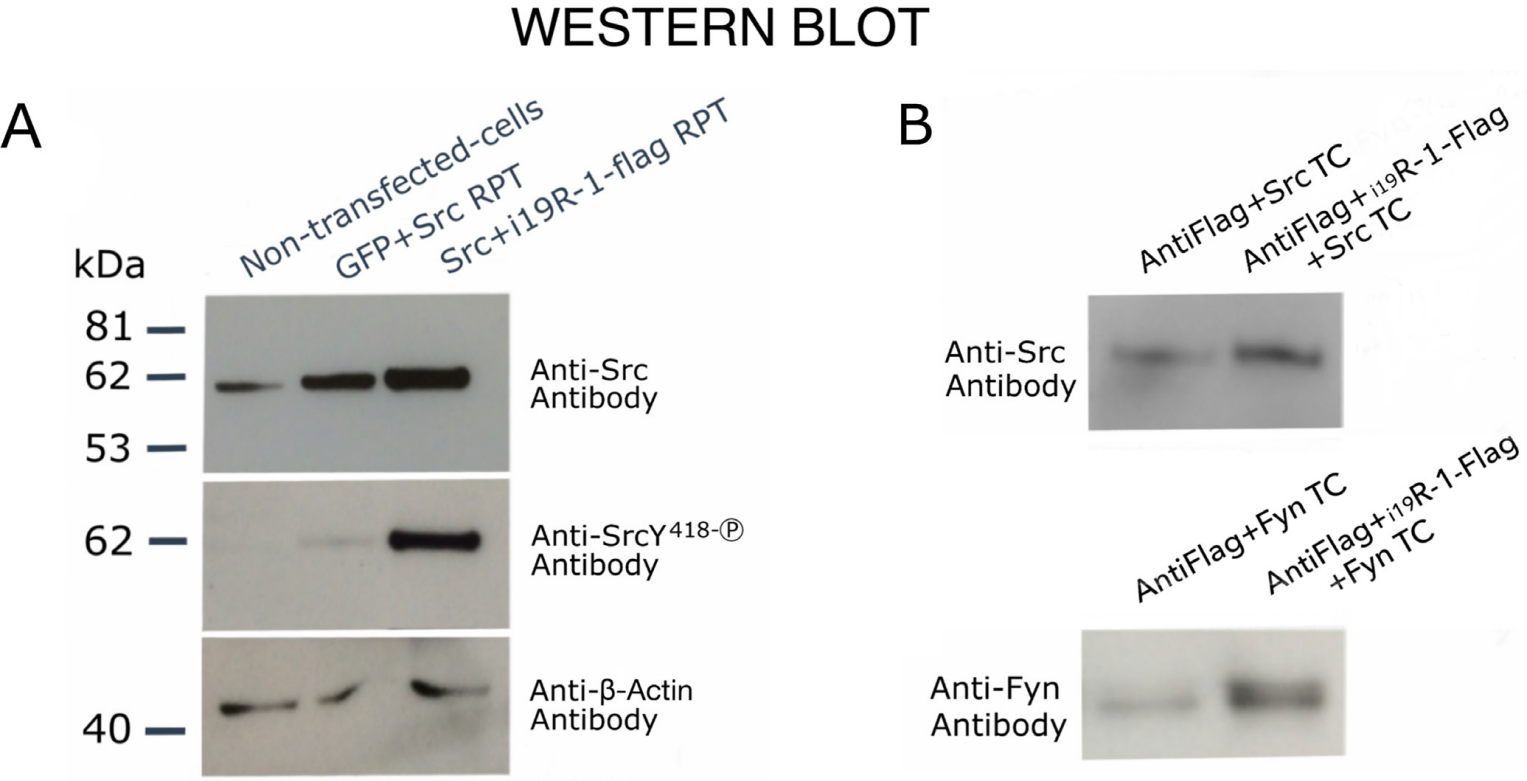
Western blot analysis showing transphosphorylating activity and binding of i_**19**_VEGFR-1 on Src kinases. (**A**) Cells (CHO-cells) were either, non-transfected, transfected (TC) with recombinant GFP plus Src recombinant plasmid (RPT), or transfected with Src recombinant plasmid (RP plus flag-tagged i_19_VEGFR-1 RP). The detecting antibodies, anti-Src, Anti-SrcY418-P (phosphorylated activating Y residue) and anti-β-Actin, are shown at right. The β-actin was used as the loading control. (**B**) Binding experiments to assess physical interaction between i_19_VEGFR-1 and SRC (FYN) kinases. Either, protein extracts over-expressing only active SRC or FYN kinases (TC, Transfected cells) or one of this kinases plus i_19_VEGFR-1 tagged to flag, were assayed for binding to and Anti-flag gel as described (see methods). Detecting antibodies are shown at left.

We have previously reported that i_21_VEGFR-1 in MDA-MB-231 breast cancer cells was able to enhance phosphorylation of Src [20]. Because the i_19_VEGFR-1 is slightly larger and contains all the i_21_VEGFR-1 domains, we reasoned that it too would enhance Src phosphorylation. Co-transfection of CHO cells or HEK293 cells with recombinant plasmids containing the coding sequences for i_19_VEGFR-1 and Src, produced a remarkable phosphorylation of tyrosine Y^418^, and so activation of SRC (Figure 6A). The increase in Y^418^ phosphorylation is much more lower when Src is transfected without i_19_VEGFR-1.

Moreover, after over-expressing Flag tagged-i_19_VEGFR-1 with Src (or Fyn) kinase by cell co-transfection we detected more Src (or Fyn) protein bound to the anti-flag resin beads when Flag tagged-i_19_VEGFR-1 protein was present (Figure 6B), suggesting binding of i_19_VEGFR-1 to Src and Fyn kinases.

### Sequence elements that may induce i_19_VEGFR-1 and i_28_VEGFR-1 transcription

The two isoforms, i_19_VEGFR-1 and i_28_VEGFR-1, originate at an alternative transcriptional start site (aTSS) in introns 19 and 28, respectively, of the full-length VEGFR-1 receptor.

**Figure 7:**
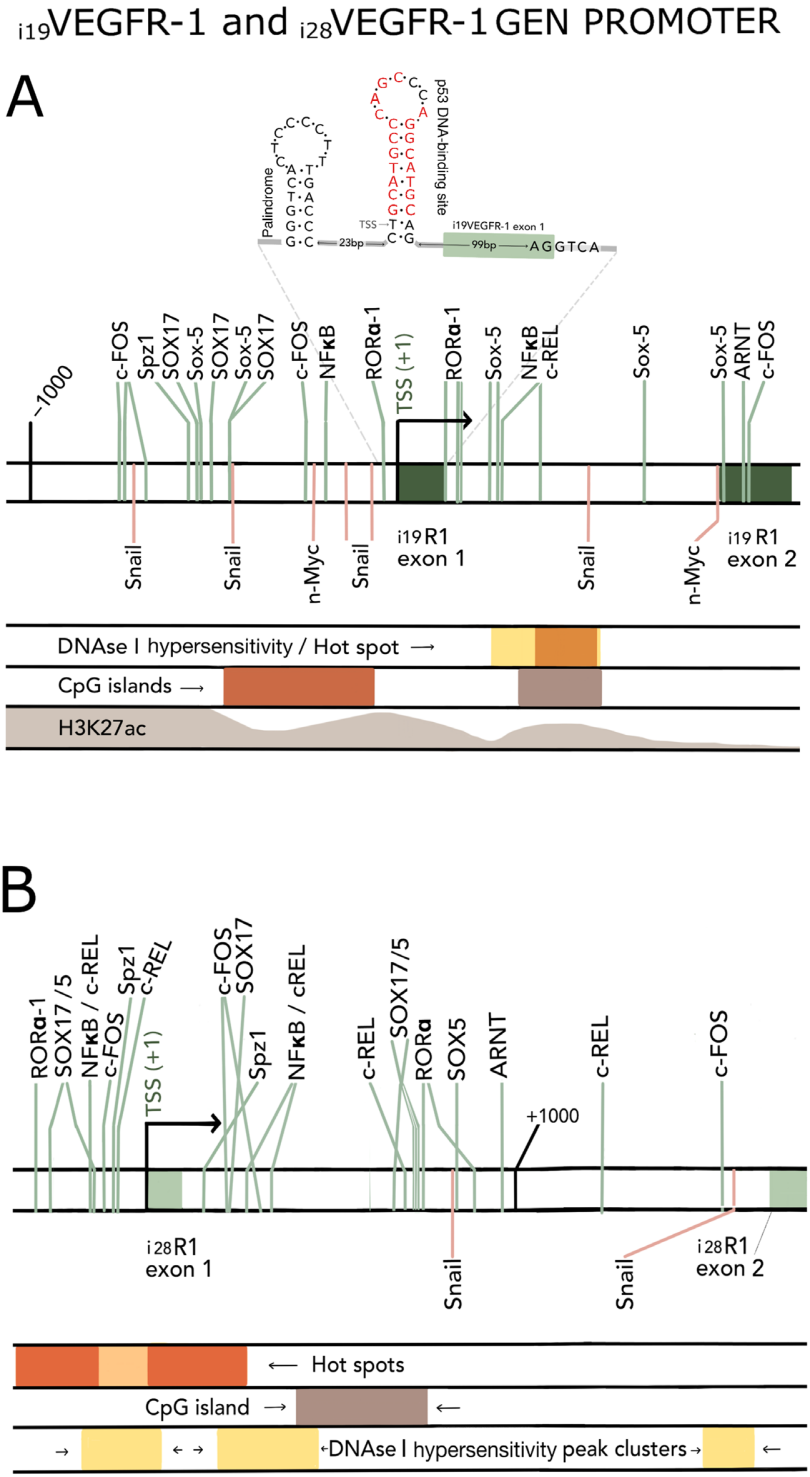
(**A**) *In silico* reported elements around the TSS (arrow) of i_19_VEGFR-1 gene. Two palindromic sequences, spaced by 23 nucleotides, first one with the stalk of the RGGTCA sequence (see results) and the second one with a p53 responsive element (RE) [22] (in red), are depicted at the top of the Figure. The p53 palindrome is just placed at the transcription start site (TSS) of i_19_VEGFR-1. Vertical lines depict DNA response elements, located in the proximal promoter and first intron. DNase I hypersensitivity hot spots (rectangles in first intron), CpG islands (rectangles in proximal promoter and first intron) and H3K27ac marks (waves decreasing in height towards the first i_19_VEGFR-1 intron) are highlighted in color. (**B**) *In silico* reported elements around the TSS (arrow) of i_28_VEGFR-1 gene. Vertical lines depict DNA response elements located in the proximal promoter and first intron. Location of hot spots (consecutive rectangles around the TSS), CpG island (rectangle in the first intron), and DNase I hypersensitivity peak clusters (rectangles around the TSS and at the end of the i_28_VEGFR-1 first intron) are highlighted in color.

We took advantage of the huge amount of available information (Encode/Jaspar/Consite) and searched *in silico* for potential DNA binding elements and chromatin cues that allowed i_19_VEGFR-1 and i_28_VEGFR-1 transcription in testis (Figure 7). The sequence RGGTCA, to which CREM factors, nuclear factors and RORα transcription factors can bind, was over-represented relatively to RCGTCA, the canonical half-CRE site [23]. A putative p53 responsive element [22] (marked in red in Figure 7A), with a 9 nucleotides stalk and 7 nucleotides loop is located just after the TSS of the i_19_VEGFR-1 gene, and is preceded (23 nucleotides before) by another palindrome with the stalk of the RGGTCA sequence just mentioned (Figure 7A).

The transcription start sites, Hot Spots for transcription activation, CpG islands, DNase hypersensitive peak clusters, and Histone H3 Lys27 acetylation (H3K27ac) marks around the aTSS of i_19_VEGFR-1 and i_28_VEGFR-1 are shown in Figure 7. Worth mentioning, are binding sites for Sox17 and Sox5, pioneering transcription factors involved in post-meiotic remodeling and over-represented near the aTSS of the i_19_VEGFR-1 and i_28_VEGFR-1. Also the Spz1 transcription factor, located in both i_19_VEGFR-1 and i_28_VEGFR-1 promoter regions, that functions as a spermatogenic Zip Protein relevant for spermatogenesis.

## DISCUSSION

Two transcript variants, i_19_VEGFR-1 and i_28_VEGFR-1, of the VEGFR-1 receptor, missing the extracellular and transmembrane domains and therefore intracellular and independent of ligands, are predominantly expressed, over the complete transmembrane transcript and soluble variants of the VEGFR-1 gene, in mature testis and human sperm cells. The two isoforms start TATA-less transcription in introns 19 and 28, respectively, in rich polypyrimidine stretches, a characteristic shared by transcripts without a TATA-box [24] and incorporate leader sequences from the respective introns were transcription starts. The palindromic p53 element [22] in the i_19_VEGFR-1 transcripts (nucleotides 2 to 22) may confer regulatory potential after transcription. The presence in the i_19_VEGFR-1 transcript of out-of-frame uORFs in the 5′UTR (Figure 1), may also modulate translation and stabilize the transcripts [25, 26].

We have previously observed that several genes selectively expressed during spermatogenesis use alternative transcription start sites and alternative splicing, giving raise to differential 5′ and 3′ regions. Also shorter 3′UTRs, by eliminating destabilizing sequences, give rise to more stable transcripts [3, 4]. In the case of i_19_VEGFR-1 and i_28_VEGFR-1 isoforms, shorter 3′UTRs are predominantly expressed (Figure 2). Shortening of the 3′UTR in VEGFR-1, eliminates a distal GT-repeat sequence of the VEGFR-1 receptor that is considered a determinant of allele instability [27].

Cryptorchid testis, having just spermatogonia germ cells and lacking differentiated spermatogenic cells, do not express i_19_VEGFR-1 and i_28_VEGFR-1 isoforms. In addition, both isoforms are enriched in spermatozoa relatively to mature testis.

We found a statistically significant (*p* < 0.0003) higher expression of i_19_VEGFR-1 mRNA in the spermatozoa of men with normal semen parameters [21] compared to asteno, oligo or oligoastenozoospermic patients, suggestive of a role for i_19_VEGFR-1 in male fertility; however, we are cautious and larger clinical studies are to be done to confirm these data.

The i_19_VEGFR-1 transcripts translate to a protein of 49kD (Figure 5). The i_19_VEGFR-1 protein is a little bigger, but similar in domains′ conservation to the isoform expressed in cancer cells [20]. Both truncated kinases activate the Src protein kinase and are very similar to Tr-KIT, a truncated variant of the c-KIT protein kinase, essential for sperm DNA integrity and proposed as a marker of human sperm quality [28], also important for activation of the parthenogenesis [29]. The i_19_VEGFR-1 kinase matches the Tr-KIT as it also conserves the phosphotranspherase-domain and the carboxy-terminal tail of the full-length protein. Without the ATP binding site, it lacks autophosphorylation ability. Anyway, i_19_VEGFR-1 induces a robust phosphorylation and activation of the Src kinases, as we have demonstrated through cotransfection of cells with both recombinant constructs (Figure 6).

Haploid expression depends on short stretches located close to the aTSS [30, 31], and truncation and the utilization of cryptic intragenic promoters has been proposed as a transcriptional signature of the germ cells undergoing terminal differentiation [5, 30]. This frequently involves the use of transcription factors like CREMτ (cAMP response element modulator tau) and GCNF (germ cell nuclear factor). These factors use an overlapping DNA binding site that is sufficient to direct cell type-specific expression *in vivo* [23, 32]. The same binding element is used by the transcription factor RORα [33] and is present several times in both i_19_VEGFR-1 and i_28_VEGFR-1 regulatory region (Figure 7). A palindromic p53 conserved DNA binding element in i_19_VEGFR-1 is not only found in the gene, it is also incorporated within the first 23 nucleotides (from 2 to 22) of the transcript and may form a hairpin structure (Figure 7A). Manual and *in silico* analysis of the regions around i_19_VEGFR-1 and i_28_VEGFR-1 aTSS, demonstrated an extensive repertoire of DNA binding sites, hot-spots, high density CpG Island, DNase hypersensitive peak clusters, and protein marks in both genes (Figure 7). These DNA regions may allow for chromatin opening and expression of the mRNA in testis cells. Worth mentioning are the presence of specific DNA binding sequence elements in the proximal promoters of these isoforms, like Sox17, Sox5 or Spz1 binding sites (Figure 7). Sox5 has been found within the nucleus of mouse post-meiotic round spermatids [34]. Sox17 is detected in about 10% of all testis specific genes [35], and Spz1, a spermatogenic Zip regulatory Protein [36], is drastically reduced in meiotic cells of oligozoospermic infertile men [37].

Given the conserved prevalence of truncated isoforms, preferentially truncated tyrosine kinases [5], within the spermiogenic transcriptome, truncated receptor kinases i_19_VEGFR-1 and i_28_VEGFR-1 may play a relevant role in and after the post-meiotic stages of spermatogenesis. From the drastic remodeling processes that the cells undergo to become spermatozoa until the sequential remodeling of the plasmatic membrane that occur during activation and capacitation. Important players in these processes are different truncated kinases [5] as well as the SRC-family kinases (SFK) [38], that are activated by selective tyrosine phosphorylation. In this processes, i_19_VEGFR-1 may be either absolutely required or play an important redundant role. Worth mentioning is the parallelism that exist between i_19_VEGFR-1 and Tr-KIT-receptor tyrosine kinases. Both belong to the same subclass (Subclass III) of receptor tyrosine kinases [39], activate SKFs, and exist in spermatozoa as truncated variants of the full transmembrane receptor [28]. Although the VEGFR-1 homozygous mice defective for the tyrosine kinase domain reaches the second generation [40], we believe that the lack of these isoform is a risky situation for the correct process of fertilization.

Potential mechanisms to explain why the expression of the i_19_VEGF isoform may be repressed in infertile semen samples, may be found at the genome level or at the stability of the mRNA. At the genome level, either, mutations in the enhancers or promoters of pioneers or other specific transcription factors, may affect negatively the expression program that develops during differentiation of spermatogenic cells, decreasing the levels of truncated isoforms like i_19_VEGF. In addition, mutations in the coding regions of the same factors, implied in TSS selection and splicing may affect their ability to produce these alternate isoforms.

In conclusion, the two i_19_VEGFR-1 and i_28_VEGFR-1 isoforms, by taking advantage of the intron-exon architecture of the VEGFR-1 gene and the opening of intronic platforms for transcription, are the predominant VEGFR-1 isoforms expressed in spermatogenic cells. They can be expressed as proteins without any dependence on ligands, and in the case of i_19_VEGFR-1, it manifests a strong ability to phosphorylate and activate Src kinases, critical for sperm capacitation and fertility. Altogether, i_19_VEGFR-1, either critical or redundant to other truncated kinases like Tr-KIT, may be an important device for sperm remodeling, capacitation and fertilization. Further clinical studies are needed to extend the significance of these VEGFR-1 isoforms as markers of human infertility.

## MATERIALS AND METHODS

### Semen samples and cryptorchidic testis

Samples were obtained after informed consent, from sperm donors and patients from the Andrology Unit of the Hospital Clinic of Barcelona, and classified as normozoospermic, astenozoospermic, oligozoospermic or oligoastenozoospermic according to WHO 2010 parameters [21]. A total of 31 samples were individually processed to obtain total RNA, then reverse transcribed and quantified by real-time PCR to analyze expression. A total of 15 samples where considered defective, in number or linear motility as just mentioned, and grouped for statistical analysis to be compared to a pool of 16 non-defective samples (named normal) (Figure 4) by the same parameters. The pool of defective samples comprised: 10 astenozoospermic (A), 2 oligozoospermic (O) and 3 oligoastenozoospermic (OA) samples.

Semen was processed as described by De Mateo et al., 2007 [41]. Cell count was carried out with a Neubauer chamber.

Cryptorchidic testis was obtained from a patient undergoing surgical preventive intervention. The histological study of this cryptorchidic testis indicated complete absence of differentiated spermatogenic cells with presence of only somatic cells and spermatogonia.

### RNA/cDNA, electrophoresis, northern hybridization, and probes

Human normal testis total RNA was obtained from Clontech (mixed population of men aged 14-60). Two different batches were tested. The cDNA was prepared from this RNA, either, by reverse transcription (SuperScript III Reverse Transcriptase cat. no. 18080-093) or by One-Step RT-PCR (Qiagen cat. no. 210212).

Human total spermatozoa RNA (see Ethics statement) was prepared with the TriPure Isolation reagent (Roche), and/or RNeasy silica-membrane spin-columns (Qiagen) following the manufacturer instructions.

Cryptorchidic testis was immediately frozen and reduced to a homogenous powder (mortar) in liquid nitrogen. Total RNA was extracted with TriPure Isolation reagent, followed by further purification in RNeasy silica-membrane spin-columns (Qiagen), as before.

The procedure used for electrophoresis, northern blot, and preparation of DNA probes has been previously described [20].

Human VEGFR-1 kinase-domain primers (Table 1) were used to obtain a probe that hybridized with the full-length VEGFR-1 mRNA and any VEGFR-1 containing the whole or part of the kinase domain and C-tail.

### RACE5′ and RACE3′

The Human Testis Marathon-Ready cDNA (Clontech cat. no. 639314) for rapid amplification of cDNA ends (RACE), the primers included in the kit and specific ones for the specific isoforms (ThermoFisher Scientific, Table 1), as well as the conditions recommended by the manufacturer, were used.

### DNA sequencing

Sequencing was conducted with the BigDye Terminator v3.1 (Applied Biosystems), as previously described, [20] and analyzed at the Autonomous University of Barcelona (CRAG Genomic Centre).

The nucleotide sequences of i_19_VEGFR-1 and i_28_VEGFR-1 isoforms were deposited in the GenBank database under accession numbers JF509744 and JF509745.

### Recombinant clones, cloning, electro-transformation, and DNA preparation

Human Src recombinant clone was from OriGene Technologies and contained v-Src sarcoma transcript variant 1 (NM_005417) in pCMV6-XL4. Human Fyn-recombinant clone was also from OriGene (NM_002037).

The i_19_VEGFR-1 coding region followed by the flag-tag were inserted into the pcDNA3.1(+) vector (Invitrogen) after enrichment by amplification from human testis cDNA with the primers: 19EAT, HFFP and HFFFA (Table 1). Restriction enzymes sites as well as the flag coding sequence, as indicated in Table 1 preceded the specific sequences in the primers. The inserted i_19_VEGFR-1 coding region was then flanked by specific restriction sites, for polarity cloning into the vector, and tagged with the Flag sequence. The recombinants having the expected insert size, were confirmed by sequencing, and high quality DNA was prepared (Qiagen) for subsequent eukaryotic transfection.

### RT-PCR and quantitative real-time PCR

Total RNA was reverse transcribed to cDNA with the iScript cDNA synthesis kit (BioRad) and specific primers, random nanomers or anchored oligo(dT). Manual (Ecogen) and semi-quantitative RT-PCR, was as previously described [20]. Real-Time PCR was performed with an ABI 7500. Specific primers expanding at least one exon were used (Table 1). Experiments where performed in duplicated, and repeated three times.

### Statistical analysis

Values are expressed as mean and standard deviations. Comparisons were made by the Wilcoxon test.

### Antibodies, SDS-PAGE, and western blotting

Antibodies and blocking peptide. Anti-Flt-1 #2893 (Cell Signalling); anti-Flt-1 (C-17): sc-316 (Santa Cruz) (blocking peptide, sc-316P Santa Cruz); anti-Src #2108 (Cell Signalling); anti-phospho-Src (pY418) 44-660G (Invitrogen/Thermo Fisher Sc); anti-Fyn antibody FYN3 #sc-16 (Santa Cruz); anti-β-actin A5316 (Sigma) (loading control); anti-α−tubulin T6074 (Sigma) (loading control). See Table 2 for antibodies’ additional details.

### Western blot

Cells were collected 48 h after transfection and lysed in lysis buffer, subjected to NuPAGE® Novex® Bis-Tris gels and NuPAGE® MES SDS gels, blotted in Nitrocellulose membranes (ThermoFisher) and hybridized with specific antibodies as recommended. Results were confirmed after performing three independent experiments.

Total human normal testis protein was from Clontech, pooled from 18 adult males aged 19-64. Semen samples were extracted for protein using Chaps or 0.5% Nonidet P-40 lysis buffer.

Peptide competition. Anti-Flt-1 Antibody (Santa Cruz) was diluted in tris-buffered saline (TBS) with or without the competing peptide at 200M fold excess as recommended (Santa Cruz).

### Binding experiments

Cells. CHO and HEK293 Cells (Dr. Carles Enrich, Faculty of Medicine, UB), HUVEC Cells (Dr. Teresa Royo, Faculty of Medicine, UB) were cultured in RPMI-10% FBS, DMEM-10% FBS or supplemented Medium 200 (Gibco) respectively.

DNA constructs transfection. CHO cells or HEK293 cells were seeded in 100 mm culture plates (Corning) at a density of 3.5 × 10^6^ cells/plate and cultured with RPMI-10% FBS. Transfections were performed with Lipofectamine® 3000 Reagent (ThermoFisher) and 5 µg of each recombinant DNA. GFP transfected cells were used as a control.

Affinity gel binding and sedimentation. Anti-Flag (DYKDDDDK) affinity gel from Biotools was used for protein capture and interaction analysis, as recommended. Briefly, proteins were extracted with 1% Triton X-100, and 500 μL were allowed to interact with 10 μL of uniformly resuspended anti-Flag affinity gel in 600 μL Tris-buffered saline (TBS), for 2 h at 4°C and with gently agitation. Then, the resin was collected by centrifugation (30 sec at 4,000xg) and washed (3–5 times) with 0.5 mL of TBS till OD280 was <0.05. Elution was carried with 2xSDS PAGE, boiled for 5 min and 4 μL loaded in SDS-PAGE gels. The experiment was reproduced 3 times with independent samples.

### *In silico* analysis

VEGFR-1 genomic sequences were analyzed for potential transcription factor binding sites using the JASPAR software [42] for single sequences and Consite software [43].

CpG islands in the VEGFR-1 gene were predicted using DBCAT (DataBase of CpG islands and Analytical Tool) software [44].

DNaseI hypersensitivity in the VEGFR-1 locus was analyzed comparing against the ENCODE genomic region derived from assays in the cell line NT2-D1 cell [NTERA-2 cl.D1 [NT2/D1] (ATCC^®^ CRL-1973) from testis, with the data deposited in the GenomeBrowser [45].
